# The impact of cognitive reserve in the recovery of chronic encephalopathy associated with traumatic brain injury

**DOI:** 10.25122/jml-2022-1007

**Published:** 2022-06

**Authors:** Silvina Ilut, Iulia Vadan, Dafin Muresanu

**Affiliations:** 1 Department of Neuroscience, Iuliu Hatieganu University of Medicine and Pharmacy, Cluj-Napoca, Romania; 2 RoNeuro Institute for Neurological Research and Diagnostic, Cluj-Napoca, Romania

## COGNITIVE RESERVE

### Definition

The onset of chronic traumatic encephalopathy (CTE) symptoms varies from person to person, with the cognitive reserve (CR) being considered one factor that may influence the age of onset. CR is defined as “the adaptability that helps to explain differential susceptibility of cognitive abilities or day-to-day function to brain aging, pathology or insult” [1]. It is considered that different processes (such as relevant lifetime exposures, education, leisure activities, occupational achievements) can influence a person's CR, not just fetal development or genetic factors. If the patient exhibits symptoms of CTE, the long-term rehabilitation continues to minimize its impact on the patient's life and his family. In conclusion, cognitive reserve and rehabilitation in CTE require future studies to fully understand the pathophysiological mechanisms.

### CR hypothesis

Every interaction changes the efficiency and flexibility of our brain networks. As a result, someone's brain resiliency and handling of aging and brain pathologies are directly influenced by their lifetime experiences [2, 3].

It is thought that CR modifies how the brain responds to preordained factors once they are present but does not act as a protective factor against different diseases [4]. Previous studies established the important role that CR plays in the outcome of Alzheimer's disease, vascular injury, Parkinson's disease, traumatic brain injury, HIV, and multiple sclerosis [5].

There are epidemiological data that support the CR hypothesis. These include the higher risk of dementia in persons with lower education and occupational attainment. In addition, persons with higher educational and occupational achievements that develop Alzheimer's disease have a more rapid cognitive decline than those with lower achievements [6].

### Cognitive reserve in traumatic brain injury (TBI) and CTE

An essential aspect of the cognitive reserve is that the clinical changes and sequelae after a brain lesion vary in relation to this reserve.

Furthermore, a brain lesion will usually affects the brain's structure from a physical and functional point of view. Therefore, the ability of the brain to adapt, through plasticity and elasticity to the pathological lesions comprising the brain and cognitive reserve, is associated with better outcomes.

A study published in 2021 by Pettemeridou et al. analyzed the correlation between the cognitive reserve and outcomes in males with TBI. The cognitive reserve was evaluated using a vocabulary test, pseudowords task, and Glasgow Outcome Scale-Extended. The consequence of the TBI on males was that TBI causes a progressive decline that interferes with semantic knowledge [7].

Venkatesan et al. studied the cognitive reserve of aging chronic TBI patients using a test based on reading different words and showed a limitation in using CR [8].

Vervoordt highlighted the impact of EPA and hippocampal reserve in the onset of depression in elderly TBI patients unrelated to cognitive decline [9].

Different methods were used to quantify the brain and cognitive reserve, for example, evaluating the brain's structure (*e.g*., volume or metabolic brain quantification).

**Figure 1:**
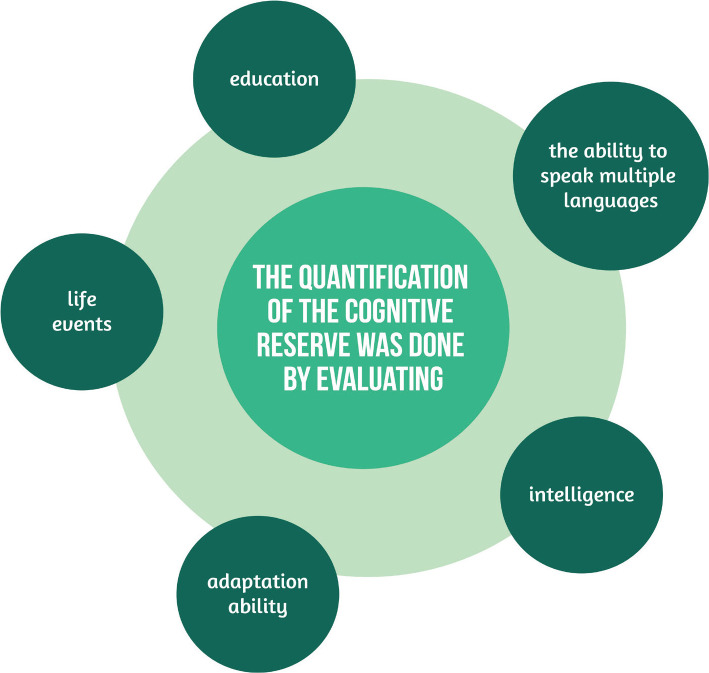
The quantification of cognitive reserve.

In contrast, the quantification of the cognitive reserve was done by evaluating:

- Intelligence;
- Education;
- The ability to speak multiple languages;
- Life events;
- Adaptation ability, as seen in Figure 1.

Many of the CR variables are intercorrelated. For example, childhood IQ, educational attainment, and occupation in middle age were studied by Stern et al. The results suggested that even if childhood factors are highly significant in the formation of CR, it is not fixed in childhood. Still, it continues to be influenced throughout life by events and circumstances that unfold before someone [10].

Recent research concentrates on the role of CR and its influence on the manifestation of CTE symptoms. Thus, higher education plays a vital role in maintaining excellent cognitive function and recovery after a TBI [11].

Outcomes after a TBI differ for every patient, which is why more and more scientists have started applying the CR theory in these cases. It is contemplated that when neuropathological damage occurs in the same manner between two individuals, the one with the higher CR will have fewer cognitive/behavioral and functioning impairments than the other [12].

In their study, Alosco et al. suggested that a higher CR was associated with the onset of cognitive and behavioral symptoms more than ten years later in a group of pathologically-confirmed cases of CTE. Moreover, the effect was even more significant in the case of behavioral symptoms of this disease.

PET and fMRI scans examined different brain regions related to solving different tasks in young adults. Still, the results were incongruent, suggesting a need to identify neural capacity and efficiency in the same task as a function of CR in young people [10].

The presence of more active models of CR suggests that the brain actively compensates for the brain damage that appears during different pathologies by making better use of the neurons which are still available [13].

### Rehabilitation

In the mid-20^th^ century, scientists decided to walk away from the assumption that the effect of a brain lesion on function, activity, and participation is permanent and become more and more aware and interested in the brain's potential to regenerate over time (months and even years). The race for finding the optimal conditions for brain change and recovery started and is still going on today.

It is well-known that disability due to neurological pathologies is rising worldwide. Disability is a burden on healthcare systems worldwide, with patients remaining with long-term psychological and functional issues. Therefore, the need for special rehabilitation services is greater than ever. Neurorehabilitation requires behavioral and functioning effort and management from diverse professions, sectors, and even family and patients.

In recent years, the mortality rates related to neurological pathology have grown significantly in lower- and middle-income countries compared to high-income countries [14].

The Center for Disease Control and Prevention (CDC) estimates that there are 1.4 million Americans seen in the hospital for a traumatic brain injury. The number of persons that suffer a TBI and do not seek medical treatment is unknown. Each year in the United States, 80000 people are left with long-term disabilities due to a TBI. At least 2% of Americans suffer from a long-term need for help for daily activities due to a TBI. In the USA, the economic impact of problems related to TBI costs more than 221 billion US dollars. Still, the total impact might be underestimated due to indirect costs that have not been considered (support services and care provided by family members) [15].

McKee et al. discovered that most cases of CTE occur in athletes, especially those practicing boxing, football, soccer, and hockey. Many of them started training in these sports between 11 and 19 years old. While the incidence of CTE is hard to establish, it is thought to vary based on sport, length of career, number of TBIs sustained during their career, age of the first head injury, and genetics [16].

**Figure 2:**
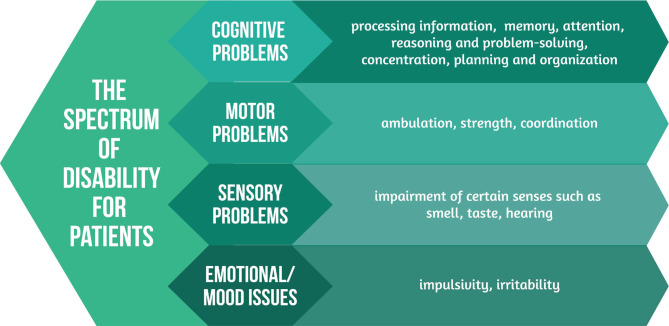
The spectrum of disability.

Moderate-to-severe TBIs need hospitalizing, and these patients frequently start rehabilitation during hospitalization to minimize their impairment level. The spectrum of disability [17] in patients is highlighted in Figure 2.

**Figure 3:**
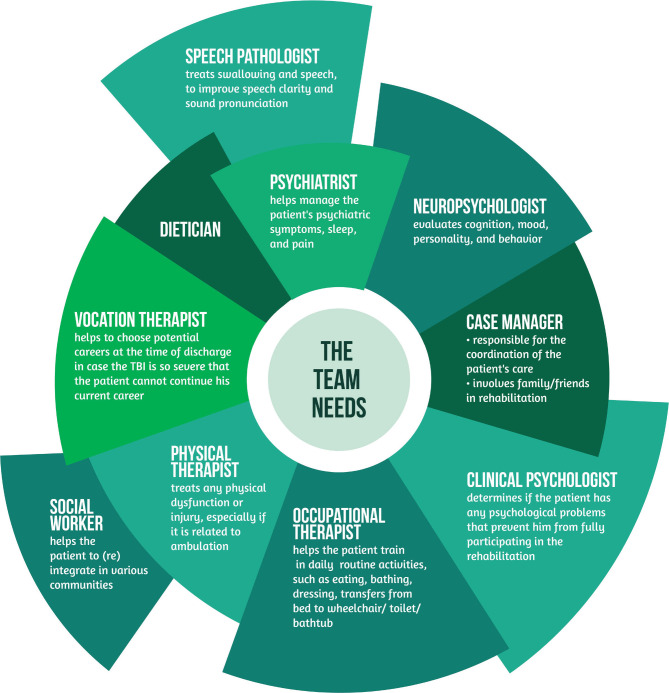
The specialists involved in neurorehabilitation.

The primary goals of every rehabilitation program should be to maximize function and minimize disability. Also, it should minimize the risk of complications as much as possible. That is why rehabilitation is interdisciplinary, as seen in Figure 3.

The role of family and friends should never be underestimated. They represent an excellent support team for the patient and help patients continue their rehabilitation after discharge.

Rehabilitation starts during hospitalization but is often a process that continues long after the hospital release. In most cases, there is a transition to an outpatient brain injury rehabilitation program. Studies show that behavioral and cognitive impairments are the most common problems patients report upon reexaminations.

Neurorehabilitation of CTE is a complex topic that ranges from early rehabilitation of patients with impaired consciousness to supporting and reintegrating patients into their family, social and professional environments as often as possible. Because of the variability of symptoms and sequelae after a TBI, rehabilitation should be individualized and focused on the patient's needs and deficits to obtain the best possible outcome.

## References

[1] Barrett AM, Oh-Park M, Chen P, Ifejika NL (2013). Neurorehabilitation: Five new things. Neurol Clin Pract.

[2] Stern Y, Arenaza-Urquijo EM, Bartrés-Faz D, Belleville S (2020). Whitepaper: Defining and investigating cognitive reserve, brain reserve, and brain maintenance. Alzheimers Dement.

[3] Pettigrew C, Soldan A (2019). Defining Cognitive Reserve and Implications for Cognitive Aging. Curr Neurol Neurosci Rep.

[4] Stern Y, Barulli D (2019). Cognitive reserve: in Handbook of Clinical Neurology. Elsevier.

[5] Tucker AM, Stern Y (2011). Cognitive reserve in aging. Curr Alzheimer Res.

[6] Scarmeas N, Stern Y (2003). Cognitive reserve and lifestyle. J Clin Exp Neuropsychol.

[7] Pettemeridou E, Constantinidou F (2021). The Association Between Brain Reserve, Cognitive Reserve, and Neuropsychological and Functional Outcomes in Males With Chronic Moderate-to-Severe Traumatic Brain Injury. Am J Speech Lang Pathol.

[8] Venkatesan UM, Rabinowitz AR, Bernier RA, Hillary FG (2022). Cognitive Reserve in Individuals Aging With Traumatic Brain Injury: Independent and Interactive Effects on Cognitive Functioning. J Head Trauma Rehabil.

[9] Vervoordt SM, Arnett P, Engeland C, Rabinowitz AR, Hillary FG (2021). Depression is associated with APOE status and hippocampal volume but not cognitive decline in older adults aging with traumatic brain injury. Neuropsychology.

[10] Baugh CM, Robbins CA, Stern RA, McKee AC (2014). Current understanding of chronic traumatic encephalopathy. Curr Treat Options Neurol.

[11] Alosco ML, Mez J, Kowall NW, Stein TD (2017). Cognitive Reserve as a Modifier of Clinical Expression in Chronic Traumatic Encephalopathy: A Preliminary Examination. J Neuropsychiatry Clin Neurosci.

[12] Steward KA, Kennedy R, Novack TA, Crowe M (2018). The Role of Cognitive Reserve in Recovery From Traumatic Brain Injury. J Head Trauma Rehabil.

[13] Stern Y (2003). The concept of cognitive reserve: a catalyst for research. J Clin Exp Neuropsychol.

[14] Khan F, Amatya B, Galea MP, Gonzenbach R, Kesselring J (2017). Neurorehabilitation: applied neuroplasticity. J Neurol.

[15] Oberholzer M, Müri RM (2019). Neurorehabilitation of Traumatic Brain Injury (TBI): A Clinical Review. Med Sci (Basel).

[16] McKee AC, Cantu RC, Nowinski CJ, Hedley-Whyte ET (2009). Chronic traumatic encephalopathy in athletes: progressive tauopathy after repetitive head injury. J Neuropathol Exp Neurol.

[17] Greenwald BD, Rigg JL (2009). Neurorehabilitation in traumatic brain injury: does it make a difference?. Mt Sinai J Med.

